# What supervisors and universities can do to enhance doctoral student experience (and how they can help themselves)

**DOI:** 10.1093/femsle/fnx090

**Published:** 2017-05-04

**Authors:** Dawn C. Duke, Pam M. Denicolo

**Affiliations:** 1 Researcher Development Programme, Doctoral College, University of Surrey, Guildford, Surrey GU2 7XH, UK; 2 Professor Emeritus, University of Reading, Reading, Berkshire RG6 6UR, UK

**Keywords:** postgraduate researcher, advisor, doctorate, PhD, employability, graduate programme

## Abstract

Over the past two decades, there has been a flurry of government papers and policy reports worldwide calling for increased number and diversity of doctoral researchers and a broadening of the curriculum to meet the developing needs of respective national ‘knowledge-driven’ economies. This has been followed by position papers and best practice examples of employability skills development in boundary-crossing doctoral programmes, especially in response to these initiatives. However, there is a disassociation between this ample literature expounding the new doctorate with its broader remit, inclusivity and production of ‘industry-ready’ graduates and the comparatively sparse literature on the doctoral candidates’ experiences of their programmes and career readiness. Within this review, we briefly outline international government initiatives and examples of the responses by Life Science and Biomedical doctoral programmes to address these various challenges. Furthermore, we explore the recent literature on the lived experience of doctoral researchers by examining their perception of the recent changes to the research context to make recommendations for universities and supervisors on how to better support an ever more diverse doctoral population for a wide range of career opportunities. Examples of how doctoral researchers themselves can make the best of currently available opportunities are also provided.

## Teaser

The maelstrom of change in the research and employability context impinging on the doctorate demands universities and research supervisors adapt process, procedures and ethos, and that students become increasingly proactive.

## THE CHANGING DOCTORATE

Before the late 1990s, the nature, content and process of research degrees were, generally, left to the academic realm. However, since then there has been an upsurge of government reviews and funding body policy changes, directly impacting the very nature of the degree itself (Denicolo, Duke and Reeves 2016). Internationally, there has been a drive to increase numbers and widen the diversity of doctoral candidates, as governments recognise the value to their respective national economies of highly educated doctoral graduates. Furthermore, it was acknowledged that to accommodate this vision, doctoral programmes would have to change to meet new demands (Kehm 2006). In Europe, the Bologna Declaration and the Lisbon Strategy included comment on the doctorate, calling for the European doctorate to include higher quality training, increased international mobility and preparation for a variety of career destinations outside of academia (European Ministry of Education 1999; Rodriguez, Warmerdam and Triomphe 2010). Meanwhile in the USA the ‘Re-envisioning the PhD*’* project undertook a detailed investigation into the American doctorate, criticising the length, attrition rates and employability training/support as not fit for purpose in the modern research context (Nyquist and Woodford 2000). Likewise, the White Paper, ‘Knowledge and Innovation’, identified similar problems with the Australian doctorate, highlighting both high attrition and lack of career readiness, particularly in non-academic sectors (Kemp and Kemp 1999). Thereafter, Sir Gareth Roberts’ ‘Set for Success’ report was published identifying key employability skills gaps in UK doctoral graduates, leading to the embedding of transferable skills training in the UK doctorate (Roberts 2002).

Over the last 10 years, there has been a plethora of position papers discussing changes that should be made to the doctorate, alongside considerable effort on the part of many universities and doctoral programmes to implement changes to address different aspects of the challenges identified in these early policy documents. Preparation for jobs outside of academia and the provision of high-quality careers support for doctoral researchers and for postdocs has been identified as critical for biomedical doctorates (Gibbs and Griffin 2013). As such, across Europe, the majority of doctoral researchers have some access to research and/or transferable skills training, and universities have worked to create an infrastructure to accommodate the international mobility agenda central to the Bologna Declaration (Parada and Peacock 2015). However, Hancock and Walsh (2016) identify key problems with the approach of simply adding transferable skills training on top of a typical STEM doctoral programme, arguing that experience within non-academic contexts is essential for doctoral graduates to effectively use these skills within the context of their careers (Hancock and Walsh 2016). Vanderford (2012) identifies the development of specific business skills as being of key importance and presents an American case study of the integration of these skills within a Life Science doctorate. Another programme, described by Porter and Phelps (2014), integrates non-academic working within the doctoral process, specifically tailoring their intersectoral experiences to match the researchers’ desired career pathways. Kemp, Newnham and Chapman (2012) describe an Australian solution to produce ‘industry-ready’ doctoral graduates; however, they highlight potential drawbacks of solely industrial-driven doctorate programmes as well.

Further complicating universities’ drive to enhance the modern doctorate, the various government drivers and initiatives can be in conflict with one another. Bossier and Eleftheriou (2015) highlighted the conflict that arises from policies that encourage additional training and mobility experiences for doctorates, while also requiring increasingly shorter time frames for completion. Likewise, in the USA, Schmidt, Robbins and Combs (2012) examined a programme designed to support doctoral students as they cross multiple boundaries, national, disciplinary and sectorial, throughout the course of their doctorate, identifying a definite strain on completion times. Even more worrying, despite work to create these innovative programmes designed to provide a broader skills base, recent reports continue to identify specific areas of weakness worldwide including doctoral student experience and satisfaction and researcher skill development and employability (Manathunga, Pitt and Critchley 2009; Wilson 2012; Allum, Kent and McCarthy 2014).

Therefore, while the big picture of the doctoral context has changed greatly, the doctoral students engaged in day-to-day research continue to find fault with the context, specifically their inclusion into the research culture, access to careers advice and employability skill development, as outlined below. In fact, to doctoral students, changes to university regulations and policies may be perceived simply as added bureaucracy rather than a large-scale shift in the purpose of the doctorate itself (Ashwin, Deem and McAlpine 2016).

## EXPERIENCE OF INTEGRATION WITHIN THE NEW DOCTORAL CONTEXT

As government bodies and funding agencies push for increasing numbers and diversity of doctoral researchers, it is important to understand how this larger, more diverse population integrates within their research community and culture, at the group, department and university level. Analysing the UK Postgraduate Research Experience Surveys, research culture experience has remained the lowest scoring response set since the survey's 2009 inception, with integration into the broader community particularly low (Hodsdon and Buckley 2011; Bennett and Turner 2013; Turner 2015). A similar trend was found in Australia and New Zealand (Brew, Boud and Malfroy 2017; Johnston *et al*. 2016). In general, science doctoral researchers report better integration than their social science and humanities peers, possibly due to working as part of a laboratory group (Fuhrmann *et al*. 2011). However, Gibbs, McGready and Griffin (2015) found that females and underrepresented minorities within Life Sciences were less likely to agree with the statement that they belonged to their department's intellectual and social community.

On closer inspection, these surveys demonstrate variability of integration experienced, with some feeling well integrated and others not at all. This could partly be due to the increasing diversification of doctoral students, as well as the growth in interdisciplinary study. The research of Jazvac-Martek, Chen and McAlpine (2011) and Walsh (2010) has identified international and part-time doctoral researchers (Bennett and Turner 2013), as well as those from underrepresented minority groups (Gibbs, McGready and Griffin 2015), as being more likely to feel isolated. This may be due to differences in tacit expectations of the doctorate and of supervision related to previous cultural and educational experiences (Gurr 2001; Sidhu *et al*. 2014). Furthermore, several studies have shown that, unless a programme is specifically designed to support interdisciplinary researchers, these doctoral researchers often feel excluded from the predominant research culture that is based around traditional disciplinary boundaries (Boden, Borrego and Newswander 2011; Strengers 2014). Therefore, although doctoral programmes are diversifying and providing increased opportunity to cross boundaries in anticipation of a new, more global and interdisciplinary research context, the lack of integration into a supportive research culture may continue to inhibit researchers from reaching their full potential, resulting in dissatisfaction and attrition.

## CHANGING CAREER LANDSCAPE

One of the most dramatic changes facing doctoral students today is the change in career opportunities available to doctorate holders. Traditionally, the doctorate was seen as *the* entrance qualification for an academic career. Today, however, doctoral graduates are employed across all sectors, often in research and/or management roles (Hodges, Metcalfe and Pollard 2011; Dybas 2013). Indeed, governments have explicitly anticipated that doctorate holders will move out of academia to careers in a variety of sectors (Roberts 2002; Wendler *et al*. 2010; Bogle 2014). Despite this, both doctoral students’ and supervisors’ understanding of this new career landscape is often poor, and can lead to misinformed expectations, disappointment and loss of opportunities.

Most candidates enter the doctorate with only vague career goals, instead following an intense passion for their research area/topic; nevertheless, many do enter with some notional desire for an academic career, a greater number than is feasible considering the number of vacancies (Turner 2015). Of particular concern, biomedical doctoral students appear to have little understanding of the alternative options available to them (Fuhrmann *et al*. 2011; Gibbs, McGready and Griffin 2015). Although most studies show a reducing trend in the number of doctoral students desiring an academic career as their programme progresses, there does not appear to be a corresponding increase in knowledge about the variety of alternatives available to them (Fuhrmann *et al*. 2011; Gibbs, McGready and Griffin 2015). Counterintuitively, this is happening at a time when there is an increased international demand within the private sector for highly skilled people, especially those with analytical and problem solving skills that are needed to drive research forward into innovation in products and practice (Dybas 2013; Bishop 2015; Kitagawa 2015). This indicates a need, from registration onwards, for more robust and tailored careers advice and support for doctoral students to help them take advantage of exciting potential career paths.

Currently, research shows that doctoral students lack recognition and understanding of the skills employers are seeking (De Grande *et al*. 2014; Walsh *et al*. 2015). Critically, this lack of understanding of employability skills and opportunity continues throughout postdoctoral stages (junior researchers) (Gibbs, McGready and Griffin 2015; Elvidge, Spencely and Williams 2017). Furthermore, even those who successfully acquire an academic career have reported feeling the doctorate did not adequately prepare them for the range of activities the role now entails (Sinclair, Barnacle and Cuthbert 2014). Thus, it is no surprise that doctoral researchers report low levels of satisfaction with careers support (Ateş *et al*. 2011; Turner 2015) and career readiness post-graduation (Parada and Peacock 2015).

## RECOMMENDATIONS FOR UNIVERSITIES

For universities, a worrying trend noted in this review is that despite transformations in the doctorate implemented to meet new government expectations, doctoral researchers’ perception of their student experience and their employability remains stubbornly negative, as described above. Even a programme designed specifically to enhance employability outside of academia identified a gap in doctoral students’ ability to translate skills gained during their doctorate to different contexts (Manathunga, Pitt and Critchley 2009). Furthermore, some developments seem to contradict each other, cf. requirements for increased training and international/intersectoral mobility versus timely completion (Schmidt, Robbins and Combs 2012; Bossier and Eleftheriou 2015). Moreover, supervisor role expectations remain fairly unchanged as they juggle multiple academic demands (Denicolo 2016). Therefore, there is much to do in order to truly prepare our doctoral researchers of today for the opportunities that await them tomorrow. Areas that appear to be most critical based on international drivers and research on doctoral students’ experience are as follows:

1. Transparency about course length, expectations and career outcomes for doctoral programmes, at the point of application.
2. Career support tailored for doctoral and postdoctoral researchers to prepare them for the range of career options
3. Employability skills training with opportunities to translate these skills across context.
4. The creation of inclusive and supportive research communities within universities that incorporate all stages of researcher development and encourage interdisciplinary networks.
5. Continuing professional development for supervisors so that they are better able to support the more diverse doctoral candidate population in preparation for careers in a wide variety of sectors.

Transparency of expectation is critical to increase researcher satisfaction. Mismatches between expectation and the reality of career aspiration, training content and length drive much dissatisfaction (Fuhrmann *et al*. 2011; Allum, Kent and McCarthy 2014; Bogle 2014; McDowell *et al*. 2014). Explicit statements about degree structure, length and variability alongside all training/development activities required/offered should precede enrolment with information/guidance about potential career opportunities incorporated into the study programme. Furthermore, all expectations, including requirement of training and development activities, should be stated upfront. At the same time, programmes should have the necessary flexibility to meet project and student needs ensuring that all are integrated into a realistic professional development and research project plan.

It is a challenge to get high-quality data on doctoral careers, primarily because the first destination is often a transition post. However, universities should invest in tracking their doctoral alumni's career pathways and use these data to help inform incoming candidates. However, as doctoral researchers are highly mobile, this may have to become a collaborative task across partners in the higher education sector. In the UK, Vitae has led the way in research on doctoral career destinations (Hodges, Metcalfe and Pollard 2011) showing that although most doctoral degree holders do not go into academia, they have a very low unemployment rate along with high job satisfaction. Research shows that many doctoral graduates go on to do at least one postdoctoral position before moving onto a career outside of academia (Fuhrmann *et al*. 2011; McDowell *et al*. 2014). This postdoctoral period remains a critical period wherein high-quality careers advice is necessary (Elvidge, Spencely and Williams 2017). Within each university, there should be careers support that is able to provide tailored guidance to doctoral researchers and postdoctoral fellows. Universities must invest in specifically qualified people and tailored infrastructure in order to meet the demand of researchers moving into such a variety of sectors and jobs.

Although transferable/employability skills training has now become embedded in many universities, training alone may not be enough to enable the transition from doctorate to career (Manathunga, Pitt and Critchley 2009; Parada and Peacock 2015). Increasingly, the importance of enabling researchers to have opportunities to translate their transferable skills into different contexts is important (Dowling 2015). Beyond mere course provision, a more holistic approach to inspire researchers is required that complements the degree process and does not cause unnecessary delays to degree completion (Bossier and Eleftheriou 2015). This will be a challenge but the benefits could be that newer researchers become enthusiastic ambassadors of their institutions, facilitating new partnerships and future collaborations beyond academe.

The need for an inclusive research culture is increasingly important in the new doctoral context. It is striking that when examining microbiology UK Research Excellence Framework impact case studies, the vast majority cross discipline, international and/or sectorial boundaries (http://impact.ref.ac.uk/CaseStudies/Results.aspx?Type=S&Tag=834). Therefore, as we train the next generation of researchers for careers within or outside of academia, we must help integrate them into this larger, more diverse research culture while fostering a collaborative atmosphere.

Supervisors remain key to a positive experience for doctoral candidates, despite changes that have meant a shift from the traditional apprenticeship model to a broader curriculum of development (Wisker, Robinson and Shacham 2007). Doctoral candidates look to their supervisors to help them integrate into the research culture and they value their support and advice on professional development and career progression. However, the increased diversity of the doctoral researcher population can lead to challenges and mismatches in expectations between student and supervisor (Sidhu *et al*. 2014; Bøgelund 2015; Parker-Jenkins 2016). Furthermore, although experienced in research supervision, academic staff may lack experience of the career opportunities and skills needed within this new research context, and are thus unable to provide adequate, up-to-date career support (Parker-Jenkins 2016). Therefore, it is critical that universities provide high-quality continued professional development (CPD) opportunities for supervisors alongside non-judgemental spaces where supervisors can support each other and share both challenges and best practice, so that a culture is created in which CPD is expected and valued at all levels. Universities should ensure that adequate time is allocated for supervision and that high-quality supervision is recognised and valued by the institution (Denicolo 2016).

## RECOMMENDATIONS FOR SUPERVISORS

In the present dynamically diversifying research context, supervising doctoral researchers may be one of the most challenging activities one can undertake, but also one of the most rewarding. High-quality doctoral supervision is central not only to doctoral success, but also to the student experience. The changing research climate, added degree requirements, including shorter time frames, along with a more diverse doctoral research population means that today's supervisors need a broader range of skills and abilities to support these newer researchers throughout their doctoral journey and into the next stages of their career, which most likely lie outside of academia (Denicolo, Duke and Reeves 2016). It is important for supervisors to identify and take advantage of support systems throughout the university and build their own professional skills to be able to successfully meet these challenges of diversification of research, of researchers and of their career trajectories. Of key importance for inclusive supervision in this new context is:

- Open and honest communication about expectations, with space for negotiation in order to best meet individual researcher's needs.
- Active support of their doctoral researchers’ integration into the broad research culture within the university and the discipline.
- Supportive attitude towards a range of skills development and career opportunities.
- Active participation in and commitment to their own continued professional development, enhancing their supervisory skills and their understanding of the new research context.

Increased diversity of doctoral researcher population means that supervisors will likely be supporting students from a range of cultural, international and underrepresented backgrounds. Studies show that more diverse doctoral student populations have a broader range of assumptions and expectations of the doctorate and of supervision (Gunnarsson, Jonasson and Billhult 2013; Sidhu *et al*. 2014; Bøgelund 2015; Parker-Jenkins 2016). Gunnarsson, Jonasson and Billhult (2013) found that the expectations of doctoral researchers and supervisors were often misaligned, leading to disagreements and negative student perceptions of supervision. Therefore, it is critical that supervisors are explicit in their expectations, and ensure that their doctoral researchers have understood and correctly interpreted their supervisory discussions (Parker-Jenkins 2016). It is also important that academics work to create shared understanding and common practice across departments and research areas.

Supporting and nurturing a positive and inclusive research culture within and across departments may help enhance the experience of doctoral researchers, especially those undertaking interdisciplinary research or who are from minority populations (Boden, Borrego and Newswander 2011; Gibbs, McGready and Griffin 2015). Supervisors can help support this type of research culture by facilitating and encouraging participation in seminar series, cross departmental journal clubs and interdepartmental events (Hancock and Walsh 2016; Parker-Jenkins 2016).

A further challenge to supervisors is that they are now expected to help develop doctoral candidates for a wide range of career destinations, even though they may only have had experience in the academic context. Academics tend to convey a preference for an academic career choice (Sauermann and Roach 2012). However, evidence shows doctoral graduates have a high job satisfaction rate outside of academia as well as within (Hodges, Metcalfe and Pollard 2011). Therefore, all career choices should be valued and encouraged. Although there may be an instinctive desire for a talented doctoral candidate to follow in one's footsteps to become an academic colleague and future collaborator, within the new research context maintaining strong links with talented doctoral graduates who move into industrial, policy or other sectors may be equally satisfying and professionally beneficial.

It is increasingly apparent that supervision today requires skills and knowledge that previously were not as critical. The days of working in relative isolation for an unspecified amount of time with one or a small group of researchers from similar backgrounds, all of whom were striving to become the next generation of academics, are long past (if they ever truly existed). Supervisors today need a wide range of skills to manage tight research project deadlines and empathetically manage researchers from a wide range of backgrounds, while supporting these researchers to produce innovative, often boundary-crossing research. It is unrealistic to expect any one person to naturally have all of these skills. Therefore, supervisors should be open to updating their professional skill set and to actively participate in the sharing of best practice, learning from and supporting the development of colleagues (Denicolo 2016). It is critical that supervisors do not feel they alone are responsible for all aspects of their doctoral candidates’ development and well-being, but are aware of and actively engaging with support services. This interaction will allow supervisors to better balance these new requirements and demands at the same time as enhancing student experience, by working in partnership to create safe places where supervisory practice can be explicitly shared and to build inclusive interdisciplinary communities to better support all doctoral students.

## RECOMMENDATIONS FOR DOCTORAL STUDENTS

It is important to recognise that undertaking a doctorate is not simply an extension of undergraduate or Masters’ degree studies; indeed, Denicolo and Reeves (2013) characterise the transition between these previous higher education levels and the doctorate as a state change, a metamorphosis even. This transformation is from student to fully recognised researcher, so much so that many formal documents in universities and journal articles in the UK now use the term ‘doctoral researcher’ rather than doctoral student. The title for the main people who guide that process remains as ‘supervisor’ in many countries though it might be more accurate and more transparent, if it were changed to the US version, ‘advisor’, since it denotes the more active role that doctoral researchers are now expected to play, gaining autonomy under the gradually decreasing guidance of academics.

Acknowledging this new, dynamic relationship and its impact on the progress of doctoral students, Denicolo, Reeves and Duke (October 2017) deliberately encourage and seek to inspire doctoral students to proactively grasp the myriad opportunities afforded by doctoral study and available in universities to enhance both their enjoyment and success in the doctorate, as well as their prospects for future employment. This extends the ideas presented by Denicolo and Reeves (2013) about how transferable skills, marketed effectively, can enhance employability. Drawing on this work and on Elvidge, Spencely and Williams (2017) work that bridges the gap between the doctoral and postdoctoral research, the following recommendations for prospective and active doctoral researchers emerge.

1. Anyone taking the bold leap into doctoral research should prepare well by investigating for goodness of fit of their needs and expectations with the available supervisory arrangements, the doctoral programme structure(s), the research culture of the potential venue (university) and the training opportunities provided.
2. Begin the process of becoming an independent researcher by ensuring that discussions, and then negotiations, with supervisors about expectations about the process and relationship take place early and frequently.
3. Seek out and weave into the research process opportunities for skills training, presenting, teaching, networking, publishing and so on, to facilitate development as a researcher and to enhance future career prospects.
4. Become active members of local research communities, shaping this to suit own and fellow doctoral researcher's needs.
5. Consider from the outset the potential real world benefit of the doctoral project and engage with a variety of stakeholders outside of academy, throughout the research process.

The choice available for those thinking about undertaking a doctorate is greater than ever before. It is a truly international endeavour, with universities across the world offering programmes with different requirements and opportunities to the best candidates from any country of origin. Therefore, it has never been more important for potential doctoral researchers to take the time to find the university, programme and supervisor that fits their specific personal, professional and career development needs. Talking with potential supervisors, as well as current doctoral researchers, can provide insight into different research groups and doctoral programmes. There are many more considerations than a university's name and ranking when deciding where to commit oneself to undertaking a doctorate (Denicolo, Duke and Reeves 2017). First and foremost, a good match between a candidate's specific research interests and professional development goals with a potential supervisor's expertise and approach to supervision is key. Also critical is finding a university and doctoral programme that has the structure and resources that will best enable an individual's growth and development from a novice to independent researcher.

While undertaking this journey to become an independent researcher, many implicit rules and expectations surrounding the doctorate come to light (Petre and Rugg 2010). To succeed in these new ways of working, guidance from a supervisor is critical. Supervisors are there to help doctoral researchers navigate these new research waters, but newer researchers must communicate their own unique needs and goals so that their supervisors can provide effective support. Therefore, it is necessary for supervisors and their doctoral researchers to openly discuss expectations, negotiating these to create an optimal working relationship. This partnership should provide a supportive environment so that newer researchers become increasingly independent as they gain confidence in their knowledge and ability (Gurr 2001).

With this lofty goal in mind, it is important that doctoral researchers take the driver's seat in their own development throughout the doctorate, not only communicating with supervisors about research skills and the progress of the research project, but also career plans and professional development needs. In this spirit of taking control of one's own professional development, there are several things that are beneficial to newer researchers. First and foremost, they should become aware of the skills needed to develop as a successful researcher (Denicolo and Reeves 2013), and then find the appropriate training and developmental opportunities necessary to gain these skills, whether they are offered within the home department, broader university or beyond. Becoming involved in journal clubs or writing groups can be advantageous so, if they do not already exist, it is a good idea to start one. Journal clubs and writing groups not only support the development of critical reading and writing skills, but also have been shown to positively impact researcher satisfaction and productivity through the building of supportive communities of researchers (Lee, Dennis and Campbell 2007; Maher *et al*^2008^; Thomson and Kamler 2012).

Research communities and professional support networks are critical to the development of any doctoral researcher, and therefore, integration within a variety of these communities, including, but not limited to, those within a home department is advisable. This will also combat the potential problem of isolation (Turner 2015). For those conducting interdisciplinary work, attending workshops and seminar series from the range of disciplines involved will provide breadth and expand the support community (Boden, Borrego and Newswander 2011). Within these communities, mentors can be found. Mentors need not be limited to those formally assigned, so seeking out people to provide advice and guidance outside of official supervisory teams can greatly enhance experience and success (Lee, Dennis and Campbell 2007). Mentors can come from a variety of levels, including more senior doctoral researchers, as well as backgrounds, including different disciplines and sectors; the latter being especially helpful for those with interdisciplinary research interests or exploring jobs outside of academia (Brooks, Hopkins and Pearson 2016).

Gaining an understanding of communities beyond academe can be advantageous, even when the goal is an academic career. In these times, when economics and practice are emphasised at least as much as creativity and originality, it behoves researchers, at whatever stage, to consider the impact that their research will have on theory, practice, economy and society (Denicolo 2014). Practice in explaining and justifying one's research to groups via public engagement extends communication skills, which is good preparation for any required viva voce and aids transition into new working environments. All of these skills will serve doctoral candidates well in any future endeavour they follow next.

A world of opportunity is open to people with life science doctorates; however, options require dedicated investigation. Thought about career choices should start early, engaging with university careers service and taking opportunities to meet employers and other people working in a wide variety of sectors. Engagement with employers, increasingly common in undergraduate degrees within discipline, should be pursued, whether it be through seminars, workshops, visits, placements, internships or combinations of these, as they fit into lab or fieldwork schedules. These provide better insight into other worlds of work, as well as opportunities to consider how transferable skills can be translated into new environments (Jones and Warnock 2015). Luck favours those who proactively seek out and grasp opportunities. This is as true in research life as it is in all other parts of life (Guccione 2016). A doctorate is an amazing time of personal growth, so full advantage should be taken of all it has to offer.

***Conflict of interest.*** None declared.
